# Recent Progress of Potentiating Immune Checkpoint Blockade with External Stimuli—an Industry Perspective

**DOI:** 10.1002/advs.201903394

**Published:** 2020-02-28

**Authors:** Jun Xu, Robert Saklatvala, Sachin Mittal, Smeet Deshmukh, Adam Procopio

**Affiliations:** ^1^ Sterile and Specialty Products MRL Merck & Co., Inc. 2000 Galloping Hill Rd Kenilworth NJ 07033 USA; ^2^ Discovery Pharmaceutical Sciences MRL Merck & Co., Inc. 33 Avenue Louis Pasteur Boston MA 02115 USA

**Keywords:** cancer treatments, clinical translation, external stimuli, external‐stimuli‐based therapeutics, immune checkpoint blockade, industry, preclinical models

## Abstract

The past decade has seen the materialization of immune checkpoint blockade as an emerging approach to cancer treatment. However, the overall response and patient survival are still modest. Various efforts to study the “cancer immunogram” have highlighted complex biology that necessitates a multipronged approach. This includes increasing the antigenicity of the tumor, strengthening the immune infiltration in the tumor microenvironment, removing the immunosuppressive mechanisms, and reducing immune cell exhaustion. The coordination of these approaches, as well as the ability to enhance them through delivery, is evaluated. Due to their success in multiple preclinical models, external‐stimuli‐responsive nanoparticles have received tremendous attention. Several studies report success in distantly located tumor regression, metastases, and reoccurrence in preclinical mouse models. However, clinical translation in this space remains low. Herein, the recent advancement in external‐stimuli‐responsive nanoconstruct‐synergized immune checkpoint blockade is summarized, offering an industry perspective on the limitations of current academic innovations and discussing challenges in translation from a technical, manufacturing, and regulatory perspective. These limitations and challenges will need to be addressed to establish external‐stimuli‐based therapeutic strategies for patients.

## Graphical abstract

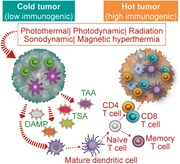

## 1 Introduction

Immune checkpoint blockade (ICB) has demonstrated unprecedented efficacy in treating various human cancers, including melanoma, urothelial carcinoma, Hodgkin's lymphoma, and non‐small‐cell lung cancer (NSCLC).^[^ 1 ^]^ Despite this, the response rate remains relatively low in most cases.^[^ 2 ^]^ Additionally, severe autoimmune‐like adverse effects limit the use of ICB for cancer treatment.^[^ 3 ^]^ Therefore, there is still a pressing need to extend cancer immunotherapy further to benefit broader patient populations. Various efforts to study the “cancer immunogram” have highlighted complex biology that necessitates a multipronged approach.^[^ 4 ^]^ To fully realize the potential of cancer immunotherapy, strategies are needed to increase the antigenicity of the tumor, amplify antitumor T‐cell immune response, remove immunosuppressive mechanisms, and reduce immune cell exhaustion.^[^ 5 ^]^

Advancement in material science has enabled the development of nanoparticles to improve the solubility, stability, and pharmacokinetic profile of cancer immunotherapy. Nanosized materials have the advantage of preferentially accumulating in solid tumors due to their hypervasculature, defective vascular architecture, and impaired lymphatic drainage—a phenomenon known as the enhanced permeability and retention (EPR).^[^ 6 ^]^ However, since only a small fraction of administered nanoparticles enter tumor tissues, the clinical relevance of EPR remains controversial.^[^ 7 ^]^ Since the liver and spleen sequester the majority of systemically administered nanomedicine, hepatotoxicity, and nephrotoxicity further hinder the clinical advancement of cancer nanomedicine.^[^ 8 ^]^ Therefore, to improve the efficacy of cancer nanomedicine and to alleviate safety issues, researchers are exploring the use of external stimuli as a strategy for next‐generation cancer nanomedicine.^[^ 9 ^]^

**Scheme 1:**
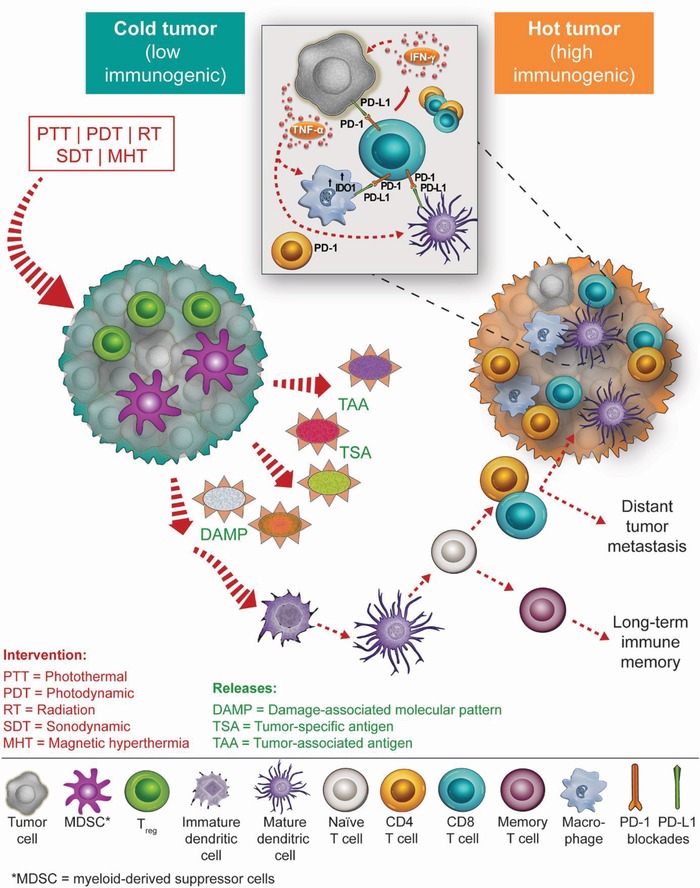
An overview of external‐stimuli‐synergized immune checkpoint blockade. Low‐immunogenic, cold tumors exhibit various immune suppression mechanisms that resist current forms of immunotherapy. External‐stimuli‐responsive nanoparticles designed for photothermal therapy, photodynamic therapy, radiotherapy, sonodynamic therapy, and magnetic hyperthermia can be used to facilitate the conversion of cold tumors into highly immunogenic hot tumors. When combined with immune checkpoint blockades, these external‐stimuli‐responsive nanoparticles could trigger a robust systemic antitumor immunological response.

When combined with external stimuli, nanoparticles have several advantages compared with conventional nanomedicine that is enabled by the EPR effect only.^[^ 9, 10 ^]^ Nanoparticles can be programmed to release cargos in response to external stimuli such as light and magnetic fields.^[^ 11 ^]^ Such controlled release can improve the therapeutic index of drugs and alleviate safety issues associated with systemic distribution. When coupled with external stimuli, such as laser irradiation and ultrasound, nanoparticles can immediately convert cold tumors into hot tumors through a direct killing effect.^[^ 12 ^]^ The direct killing effect—mediated by external‐stimuli‐responsive nanoparticles—induces immunogenic cell death (ICD) through the release of a tumor‐associated antigen (TAA) and damage‐associated molecular patterns (DAMP) which, in turn, trigger antigen presentation cell (APC) maturation, APC‐mediated T‐cell activation, and ultimately, the stimulation of immune responses against tumor cells (**Scheme** **1**).^[^ 13 ^]^ Often, immune adjuvants delivered by nanoparticles could facilitate the generation of in situ tumor vaccines to substantiate tumor‐specific immunological responses.^[^ 14 ^]^ Such tumor‐specific immunological responses could further attack the residual cancer cells, which demonstrates the potential to inhibit tumor metastasis and prevent recurrence.

In this review, we surveyed the recent progress in external‐stimuli‐responsive nanomedicine‐synergized ICB, namely photothermal therapy (PTT), photodynamic therapy (PDT), radiation therapy, ultrasound‐responsive therapy, and magnetic‐responsive therapy (**Table** **1**). We summarized recent clinical studies that demonstrate initial clinical translation feasibility and safety. Lastly, we offer an industry perspective on the field of external‐stimuli‐enabled ICB, challenges in translation (including technical implementations), and regulatory precedence. These considerations and the evolving oncology therapeutic space will need to be addressed to establish external‐stimuli‐mediated ICB as a viable therapeutic strategy for patients.

**Table 1:** Summary of external‐stimuli‐synergized ICB surveyed from 2016 to date

ICB	Cancer	Therapeutic Outcome			Ref.
		Primary tumor	Abscopal effect	Antirecurrence	
Photothermal therapy with immunotherapy					
anti‐PD‐L1, anti‐CTLA‐4	4T1, CT26	Regression	Detained growth	No recurrence	^[^ 28 ^]^
anti‐PD‐L1	4T1	Regression	Detained growth	No recurrence	^[^ 90 ^]^
anti‐PD‐1	4T1	Regression	Detained growth		^[^ 91 ^]^
anti‐PD‐L1	B16F10	87% inhibition	Detained growth	Detained growth	^[^ 23 ^]^
anti‐PD‐L1	CT26, 4T1	Regression	Regression	No recurrence	^[^ 29 ^]^
anti‐PD‐1	CT26, 4T1	Detained growth	Detained growth		^[^ 11c ^]^
anti‐PD‐L1	4T1	60% regression	88.5% inhibition	Detained growth	^[^ 26b ^]^
anti‐PD‐L1	4T1	Detained growth	Detained growth		^[^ 92 ^]^
anti‐PD‐1	B16F10	Detained growth			^[^ 93 ^]^
anti‐PD‐1	4T1	Regression	Detained growth		^[^ 94 ^]^
anti‐PD‐1	4T1, B16F10			Detained growth	^[^ 31 ^]^
Photodynamic therapy with immunotherapy					
anti‐PD‐L1	CT26	Regression	>90% inhibition	No recurrence	^[^ 44 ^]^
anti‐PD‐L1	B16F10	Near elimination		25% recurrence	^[^ 48b ^]^
anti‐PD‐L1	4T1	Detained growth	Detained growth		^[^ 49 ^]^
anti‐CTLA‐4	4T1, CT26	Regression	Regression	No recurrence	^[^ 54c ^]^
anti‐PD‐L1, anti‐CTLA‐4	GL261, CT26	Detained growth		Detained growth	^[^ 95 ^]^
anti‐CTLA‐4	CT26	Regression	Detained growth	No recurrence	^[^ 54b ^]^
anti‐CTLA‐4	MC38, CT26	Near elimination	Detained growth		^[^ 37 ^]^
anti‐PD‐L1	MC38, CT26	Regression	Regression		^[^ 96 ^]^
anti‐PD‐L1	4T1, TUBO	Regression	Detained growth		^[^ 97 ^]^
anti‐PD‐L1	B16F10	Regression		75% inhibition	^[^ 48a ^]^
anti‐PD‐L1	4T1	84.2% inhibition			^[^ 52 ^]^
anti‐PD‐L1	CT26	Regression		No recurrence	^[^ 98 ^]^
anti‐PD‐L1	B16F10	Detained growth	Detained growth		^[^ 45 ^]^
anti‐PD‐L1	B16F10, 4T1	Regression	Regression	No recurrence	^[^ 50 ^]^
Radiotherapy with immunotherapy					
anti‐PD‐L1	CT26	Regression	Regression		^[^ 71a ^]^
anti‐CTLA‐4	4T1, VX2	Regression	Regression	No recurrence	^[^ 73 ^]^
anti‐PD‐L1	4T1, CT26	Regression	Regression	No recurrence	^[^ 72 ^]^
anti‐CTLA‐4	4T1, CT26	Regression	Regression	No recurrence	^[^ 74 ^]^
Ultrasound therapies with immunotherapy					
anti‐PD‐1	CT26	Detained growth			^[^ 78 ^]^
anti‐PD‐L1	CT26, 4T1	95% inhibition	83% inhibition	Detained growth	^[^ 12a ^]^
anti‐CTLA‐4	CT26	Regression	Regression		^[^ 80 ^]^
Magnet therapies with immunotherapy					
anti‐PD‐L1	4T1, CT26	Detained growth		Detained growth	^[^ 85 ^]^
anti‐CTLA‐4	CT26	Regression	Regression	No recurrence	^[^ 86 ^]^
anti‐PD‐L1	4T1	Regression	Detained growth		^[^ 87 ^]^
anti‐CTLA‐4	MCF7, 4T1	Regression	Regression		^[^ 88 ^]^

## 2 Photothermal Therapy and Its Immunomodulatory Effect

The general principle behind PTT is the conversion of absorbed energy by photosensitizers after light irradiation into heat.^[^ 15 ^]^ Tumor cells can be destroyed by local hyperpyrexia (40–44 °C), primarily through programmed cell death.^[^ 16 ^]^ The released tumor‐associated antigens could then attract immune cells such as dendritic cells (DC), and elicit adaptive immunity.^[^ 17 ^]^ Additionally, various immunogenic responses can be triggered by elevated temperatures, including the amplification of lymphocyte trafficking into lymphoid organs, which is mediated by pyrogenic cytokine and the induction of heat shock protein expression.^[^ 18 ^]^

However, the therapeutic outcome of PTT alone remains limited by the penetration depth of light, which is ≈6 mm with a near‐infrared (NIR) laser.^[^ 19 ^]^ Although PTT triggers antitumor immunity, its effect is generally not robust enough to control established tumors with an immunosuppressive microenvironment, such as the presence of T_reg_ cells and immunosuppressive myeloid cells. Stress‐protein‐induced immunoregulatory cytokine secretion further limits the efficacy of PTT as monotherapy.^[^ 20 ^]^

### 2.1 Photothermal Therapy with Nanoparticles to Potentiate Immunotherapy

These challenges can be addressed by combining PTT with ICB and nanoparticles to synergistically induce antitumor immunity for the treatment of large established tumors. One strategy is to alter the metabolism of immune cells through indoleamine 2,3‐dioxygenase (IDO) inhibition. IDO is an enzyme that degrades tryptophan; several studies indicate that IDO induces T_reg_ cell differentiation and hyper‐activation, which suppresses effector T‐cell immune responses and decreases dendritic cell function.^[^ 21 ^]^ Peng et al. demonstrated the addition of the IDO inhibitor could induce differentiation of T cells to CD8^+^ T cells and aid in inhibiting the growth of the tumor margin as well as distal tumor growth beyond PTT.^[^ 22 ^]^ When combining PTT and IDO inhibition with antiprogrammed death ligand 1 (PD‐L1) antibodies, the treatment triggered robust immunological memory and prevented tumor recurrence.^[^ 23 ^]^ These two studies used organic photosensitizers, IR780 and IR820, respectively. Even though their photophysical properties can be precisely controlled with dedicated synthesis, they mostly suffer from photobleaching, low absorption cross‐section, poor photothermal conversion efficiency, low aqueous dispersibility, and long‐term toxicity.^[^ 24 ^]^ In contrast, inorganic nanoparticle photosensitizers offer several advantages, including resistance to photodegradation, easier functionalization, a better aqueous dispersibility, and a higher molar extinction coefficient.^[^ 25 ^]^ Studies using inorganic photosensitizers—such as reduced graphene oxide‐based nanosheets and a gold/platinum nanosystem—in combination with anti‐PD‐1antibodies achieved excellent antitumor immunity.^[^ 26 ^]^

**Figure 1:**
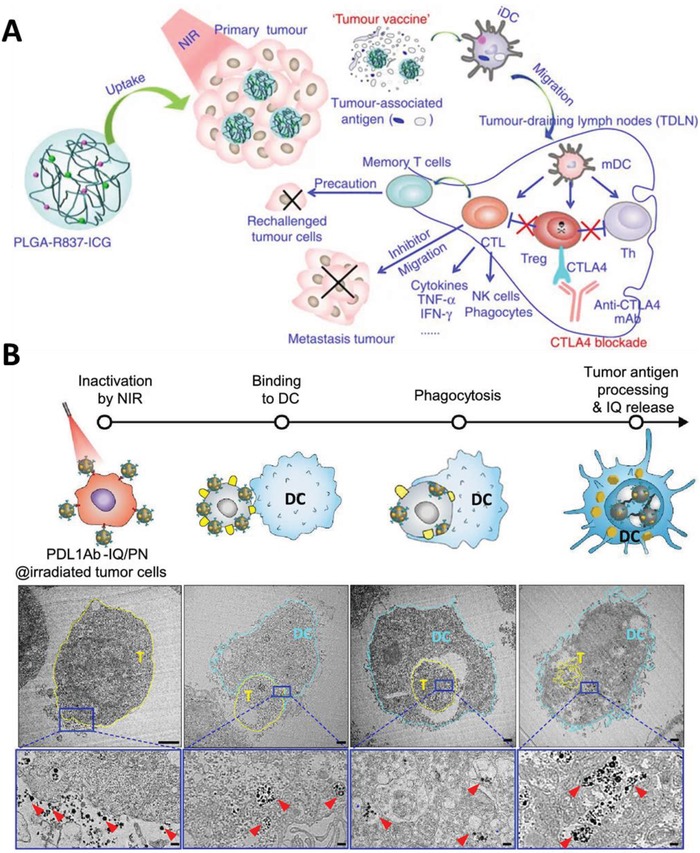
A) Schematic depiction of the mechanism of the antitumor immune response induced by TLR agonist‐based PTT in combination with CTLA‐4 blockade. Reproduced with permission.^[^ 28 ^]^ Copyright 2016, Springer Nature. B) Illustration and TEM images of DC binding, phagocytosis, and digestion of tumor cells treated with PD‐L1 antibody‐bound nanoparticles and exposed to light irradiation. The sky‐blue dotted lines indicate the shape of DCs, whereas the light‐yellow dotted lines indicate the shape of the tumor cells. Reproduced with permission.^[^ 29 ^]^ Copyright 2019, American Chemical Society.

Aside from the metabolic alteration of the immune response through IDO inhibition, researchers have pursued the use of an immune adjuvant, such as a toll‐like receptor (TLR) 7/8 agonist, to orchestrate antitumor immunity. These agonists provide essential requirements for initiating T cell‐mediated immunity, such as antigen uptake, processing and presentation by APCs, and the maturation and activation of T cells.^[^ 27 ^]^ Chen et al. demonstrated that robust immunological responses driven by improved DC maturation could be achieved by combining PTT ablation with imiquimod, a potent TLR7 agonist.^[^ 28 ^]^ Furthermore, anticytotoxic T‐lymphocyte‐associated protein 4 (CTLA‐4) antibodies that inhibit the activities of T_reg_ cells helped to inhibit tumor metastasis (**Figure** **1**A) successfully.

In a separate study, Le et al. demonstrated that coating the surface of nanoparticles containing imiquimod and photosensitizer with anti‐PD‐L1 antibody can significantly increase nanoparticle accumulation in tumors overexpressing PD‐L1.^[^ 29 ^]^ They represented a sequence of transmission electron microscopy (TEM) images that demonstrated the presence of nanoparticles on tumor cell surface sensitizes tumor cells to irradiation‐induced ICD and generates antitumor immunity (Figure 1B). This in situ‐generated tumor vaccine, when combined with additional anti‐PD‐L1 antibodies, successfully prevented long‐term recurrence for up to 150 d after the primary tumor was discovered. Through immune cell depletion assays, major cell types involved in antitumor effects include NK cells and CD8^+^ T cells, but not neutrophils, macrophages, and CD4^+^ T cells. It is worth pointing out that, in most studies, anti‐PD‐L1 or other ICB is given as a separate systemic therapy; however, in Le's study, decorating anti‐PD‐L1 antibody on the surface of a nanoparticle containing immune adjuvant served 3 functions: First, it behaved as an active tumor‐targeting ligand that enhanced the tumor accumulation of nanoparticles; second, it facilitated the formation of an in situ vaccine that enabled the codelivery of tumor antigens and adjuvants to the same APC; and third, it blocked the immune checkpoint PD‐L1 in tumor cells.

However, this approach still cannot address the “on‐target but off‐tumor” binding issue faced by anti‐PD‐L1 antibodies, which may result in severe immune‐related adverse events due to PD‐L1 expression on vascular endothelium, pancreatic islet cells, hepatocytes, and mesenchymal stem cells.^[^ 30 ^]^

In addition to the delivery of IDO inhibitors and TLR7/8 agonists, nanoparticles could also be used to deliver tumor antigens. Ye et al. utilized a surgically removed tumor to prepare a customized PTT vaccine in which a tumor cell membrane was used to coat the surface of nanoparticles.^[^ 31 ^]^ These tumor cell membrane‐coated nanoparticles were loaded into a thermosensitive hydrogel system containing the immunostimulants granulocyte‐macrophage colony‐stimulating factor (GM‐CSF) and lipopolysaccharide (LPS); both are potent dendritic cell activators.^[^ 32 ^]^ When combined with an anti‐PD‐L1 antibody, their approach successfully eliminated surgical tumor residuals and prevented tumor relapse and metastasis.

### 2.2 The Clinical Landscape of Photothermal Therapy in Combination with Immunotherapy

Despite a great preclinical proof‐of‐concept, to the best of our knowledge, there is no clinical assessment of PTT in combination with ICB. A few trials were conducted to assess the clinical benefit of PTT as monotherapy.^[^ 33 ^]^ Difficulties in the upscaling and optimization of nanoparticles and their production impose significant hurdles in their clinical translation.

## 3 Photodynamic Therapy and Its Immunomodulatory Effect

Compared to PTT, PDT is a more mature phototherapy strategy, which was approved for clinical use in 1993. The general principle behind PDT is the conversion of absorbed energy by photosensitizers after irradiation into the release of reactive oxygen species (ROS), such as singlet oxygen, hydrogen peroxide, organic peroxides, and hydroxyl anion radicals by intersystem crossing relaxation.^[^ 34 ^]^ PDT can elicit antitumor immunity by inducing necrosis and/or apoptosis in tumor cells, thereby releasing tumor antigens that provoke inflammation.^[^ 35 ^]^ Increased inflammatory cytokines and chemokines have been detected in the serum of mice after receiving PDT, including IL6, IL1β, IL8, IL10, and tumor‐necrosis factor‐α (TNF‐α).^[^ 36 ^]^ Collectively, the local inflammation triggers the infiltration of immune system cells into treated tumor areas.

Although PDT triggers antitumor immunity, its effect is as monotherapy is too weak to eradicate distant tumors.^[^ 37 ^]^ Factors that negatively affect PDT efficacy include the hypoxic nature of the tumor and poor tumor‐localization of the photosensitizers. Tumor hypoxia arises because of a decreased oxygen supply from a disorganized tumor vasculature or increased oxygen demand from changes in tumor metabolism.^[^ 38 ^]^ As the effect of almost all photosensitizers is oxygen dependent, the hypoxic microenvironment dampens the photosensitization efficiency of PDT.^[^ 39 ^]^ The half‐life of ROS in biological systems is less than 0.04 µs; thus, this very transient nature of ROS requires precise tumor localization to elicit tumor‐specific photodamage and cytotoxicity.^[^ 40 ^]^ Additionally, as a result of collateral damage to healthy cells, PDT induces immune tolerance through the release of self‐antigens and immunosuppressive cytokines, which can dampen the abscopal effect.^[^ 41 ^]^

### 3.1 Photodynamic Therapy with Nanoparticles to Potentiate Immunotherapy

To overcome the limitations of PDT monotherapy, the combination of PDT with ICB can be applied to address immunosuppression. Several unique strategies using nanoparticles can be deployed to overcome tumor hypoxia and poor tumor‐specific photodamage and cytotoxicity.

A class of nanophotosensitizers based on nanoscale metal‐organic frameworks (nMOFs) was designed to address issues faced by conventional photosensitizers, which are hydrophobic and prone to aggregation in the biological system.^[^ 42 ^]^ nMOF‐based nanophotosensitizers directly incorporate photosensitizers as the building units, allowing for high loadings without self‐quenching.^[^ 43 ^]^ Further improvements were made by Lan et al. to incorporate Fe_3_O clusters and 5,10,15,20‐tetra(*p*‐benzoato)porphyrin (TBP) into nanophotosensitizers that are capable of sensitizing PDT under hypoxic conditions.^[^ 44 ^]^ Fe_3_O can effectively decompose H_2_O_2_ to generate O_2_ via Fenton‐like reaction, whereas the generated O_2_ was converted to cytotoxic singlet oxygen (^1^O_2_) by photoexcited porphyrins. Their strategy significantly enhanced PDT efficiency and elicited robust abscopal effects in a mouse model of colorectal cancer when combined with anti‐PD‐L1 antibodies. Subsequently, the same group developed cupric ion (Cu^2+^)‐based nMOF containing TBP, capable of catalyzing estradiol‐dependent ROS generation.^[^ 45 ^]^ Studies have shown that estrogens can form stable adducts with DNA by generating ROS in the downstream metabolic processes that are catalyzed by bioavailable Cu^2+^ ions.^[^ 46 ^]^

Ni and colleagues demonstrated that locally supplied Cu^2+^ could hijack the estrogen metabolic pathways and promote cytotoxic superoxide and peroxide generation, whereas disassociated porphyrin can generate ^1^O_2_ through the PDT process.^[^ 45 ^]^ They further showed the Cu^2+^‐based nMOF, when combined with anti‐PD‐L1 antibodies, not only can induce an innate immune response but can also augment the tumor‐specific adaptive response, resulting in regression in both primary and distant tumors.^[^ 45 ^]^

To achieve higher tumor specificity and localization, as well as increase the EPR effect, tumor microenvironment responsive nanoparticles were designed. Below, we summarized dynamic targeting strategies that use functional moieties sensitive to a variety of tumor microenvironment stimuli, including pH, matrix metalloproteinase‐2 (MMP‐2), and hyaluronidases.

In contrast to healthy tissues, the extracellular pH of tumors is generally acidic, due to exacerbated glycolysis and respiration through the hydration of CO_2_.^[^ 47 ^]^ Nanoparticles with pH‐responsiveness were developed to boost tumor‐specific PDT cytotoxicity.^[^ 48 ^]^ Yang et al. incorporated a pH‐responsive charge‐reversible polymer containing dimethylmaleic acid into their nanophotosensitizers.^[^ 49 ^]^ Those nanophotosensitizers, when under pH 6.8, would undergo rapid charge conversion from negative to positive, enabling enhanced tumor cellular internalization. Because of robust INF‐γ production and significantly increased cytotoxic T‐cell infiltration, these nanophotosensitizers—when combined with anti‐PD‐L1—efficiently inhibited the primary tumor progression and achieved an abscopal effect.

**Figure 2:**
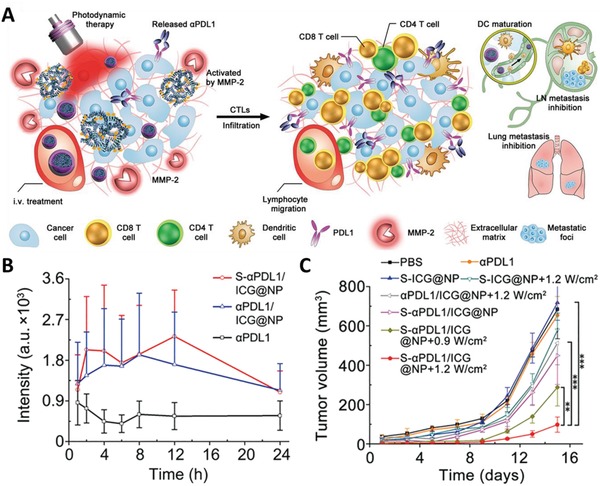
A) Schematic illustration of MMP‐2‐responsive nanoparticle‐mediated combination ICB and PDT therapy. Nanoparticles (S‐αPDL1/ICG@NP) could be activated within the tumor microenvironment for sustained release of anti‐PD‐L1 antibodies. Upon light irradiation, PDT‐induced antitumor immunity, when combined with PD‐L1 inhibition, could collectively suppress tumor growth and inhibit metastasis. B) Superior anti‐PD‐L1 antibody accumulation in the lymphatic metastasis tumors was achieved by S‐αPDL1/ICG@NP whereas negligible in the free anti‐PD‐L1 group. C) S‐αPDL1/ICG@NP + PDT at 1.2 W cm^−2^ significantly suppressed the progression of the secondary tumors rechallenged 25 d after the resection of the primary tumors. Reproduced with permission.^[^ 50 ^]^ Copyright 2019, The American Association for the Advancement of Science.

Apart from pH‐responsive nanoparticles, MMP‐2‐sensitive nanoparticles were developed to enable tumor‐specific PDT‐triggered T‐cell activation.^[^ 50 ^]^ Elevated MMP‐2 expression was reported in various cancer tissues, primarily driven by the imbalance between MMP‐2 and their natural inhibitors.^[^ 51 ^]^ Wang et al. developed MMP‐2‐sensitive nanoparticles to enable tumor‐specific delivery of anti‐PD‐L1 antibodies (**Figure** **2**A). MMP‐2 responsiveness was attributed to the incorporation of an MMP‐2‐liable proline‐leucine‐glycine‐leucine‐alanine‐glycine peptide spacer. When compared to systemically delivered anti‐PD‐L1, MMP‐2 responsive nanoparticles showed a 10.7‐fold increase in intratumoral antibody accumulation (Figure 2B). When used in conjunction with localized light irradiation that induced antitumor immunity and promoted the intratumoral infiltration of CD8^+^ T cells, these nanoparticles outperform systemic anti‐PD‐L1 in limiting growth and metastasis of murine tumors (Figure 2C).

Lastly, a hyaluronidase‐responsive, size‐reducible nanophotosensitizer was developed by Yu and colleagues to explore the size‐dependent EPR effect.^[^ 52 ^]^ They found that particles 150 nm in size, which are much bigger than those suggested by other researchers, exhibited the best tumor targeting and accumulation effects.^[^ 53 ^]^ When these 150 nm‐sized nanoparticles were loaded with photosensitizer and combined with anti‐PD‐L1 blockade, they inhibited tumor growth and metastasis.

It is worth mentioning that in addition to the strategies introduced here in Section 3, strategies presented in the earlier section (such as the codelivery of IDO inhibitors, TLR agonists, and adjuvants) were also examined by various groups. They all showed enhanced therapeutic outcomes of PDT in combination with ICB.^[^ 54 ^]^

### 3.2 The Clinical Landscape of Photodynamic Therapy in Combination with Immunotherapy

Despite considerable preclinical evidence supporting the existence of a synergistic effect between PDT and ICB, there is no active clinical trial assessing their clinical benefits. As a monotherapy, PDT has been clinically approved for treating certain cancers or precancers, including endobronchial cancer, esophageal cancer, and actinic keratosis.^[^ 55 ^]^ However, due to the limited depth of light penetration, PDT has not been approved for treating cancers that have grown deeply into the skin or other organs. Clinical assessment of PDT's immune modulation effect is currently underway (ClinicalTrials.gov Identifier: NCT03643744). The result might substantiate combining a selection of ICB candidates and nanophotosensitizers with a PDT regimen to combat cancer.

## 4 Radiotherapy and Its Immunomodulatory Effect

Radiotherapy has become the routine strategy for the management of several locally advanced, solid tumors and is now being used to treat more than half of all cancer patients.^[^ 56 ^]^ During radiotherapy, high energy ionizing radiation such as X‐rays, gamma rays, or fast‐moving charged particles could cause DNA damage, particularly DNA double‐strand breaks.^[^ 57 ^]^ Furthermore, similar to PDT, radiation can result in oxidative changes because of the continuous generation of ROS, which could further break DNA.^[^ 58 ^]^ Tumor cells are more vulnerable to radiation‐induced DNA damage than most healthy cells because they often have a DNA repair deficiency.^[^ 59 ^]^ DNA damage would result in cell death via various pathways, including apoptosis, necrosis, autophagy, or mitotic catastrophe.^[^ 60 ^]^ Additionally, radiation‐induced degradation of proteins and the release of endogenous danger signals during cell death could also induce immunogenic cell death.^[^ 56a ^]^

Studies have revealed that complementary immunostimulatory mechanisms were also activated after radiation, including the activation of the stimulator of interferon genes (STING)‐mediated DNA‐sensing pathway, and the upregulation of the MHC‐I and calreticulin expression.^[^ 61 ^]^ These additional mechanisms further activated dendritic cells and enhanced cross‐presentation of tumor antigens, thus promoting antitumor immune responses. Although antitumor immunity is induced by radiotherapy, the effect may be limited due to the presence of radioresistant suppressor cell types in the tumor microenvironment.^[^ 62 ^]^ In addition, tumor irradiation induces the expression of immune checkpoint molecules, such as PD‐L1, and upregulates TGF‐β signaling, which triggers a wound healing response. This process may facilitate tumor recurrence.^[^ 63 ^]^

### 4.1 The Clinical Landscape of Radiotherapy in Combination with Immunotherapy

Considering the mixed immunomodulatory effects induced by radiotherapy, researchers have made extensive efforts to combine radiotherapy with immunotherapy to prevent tumor metastasis and recurrence.^[^ 56, 64 ^]^ In fact, over 100 clinical trials are currently underway to evaluate the potential synergy between radiotherapy and PD‐1/PD‐L1 checkpoint blockades.^[^ 65 ^]^ CTLA‐4 inhibitors are also heavily evaluated in combination with radiotherapy.^[^ 66 ^]^ The clinically significant synergy between ICB and radiotherapy has been documented in several studies.^[^ 67 ^]^ For example, a phase 1 trial of 98 patients with locally advanced or metastatic non‐small‐cell lung cancer treated with anti‐PD‐L1 antibodies showed significantly improved median survival when treatment was combined with radiotherapy.^[^ 68 ^]^

In spite of these encouraging clinical results, unexpected treatment‐related adverse events may limit the clinical advancement of this combination strategy. In fact, in patients with bladder cancer, dose‐limiting urinary toxicity was reported when anti‐PD‐L1 antibodies were combined with radiotherapy.^[^ 69 ^]^

### 4.2 Radiotherapy with Nanoparticles to Potentiate Immunotherapy

To fully realize the potential of integrated radioimmunotherapy, various nanoparticles were developed aiming to alleviate the safety concern of such an approach and to improve the efficiency in radiosensitization. The use of heavy metal‐based nanoparticles, such as HfO_2,_ has been shown as promising radioenhancers. Heavy metal‐based nanoparticles have higher X‐ray absorption coefficients, and even low doses can achieve significant radiosensitization when deposited into tumors.^[^ 70 ^]^

**Figure 3:**
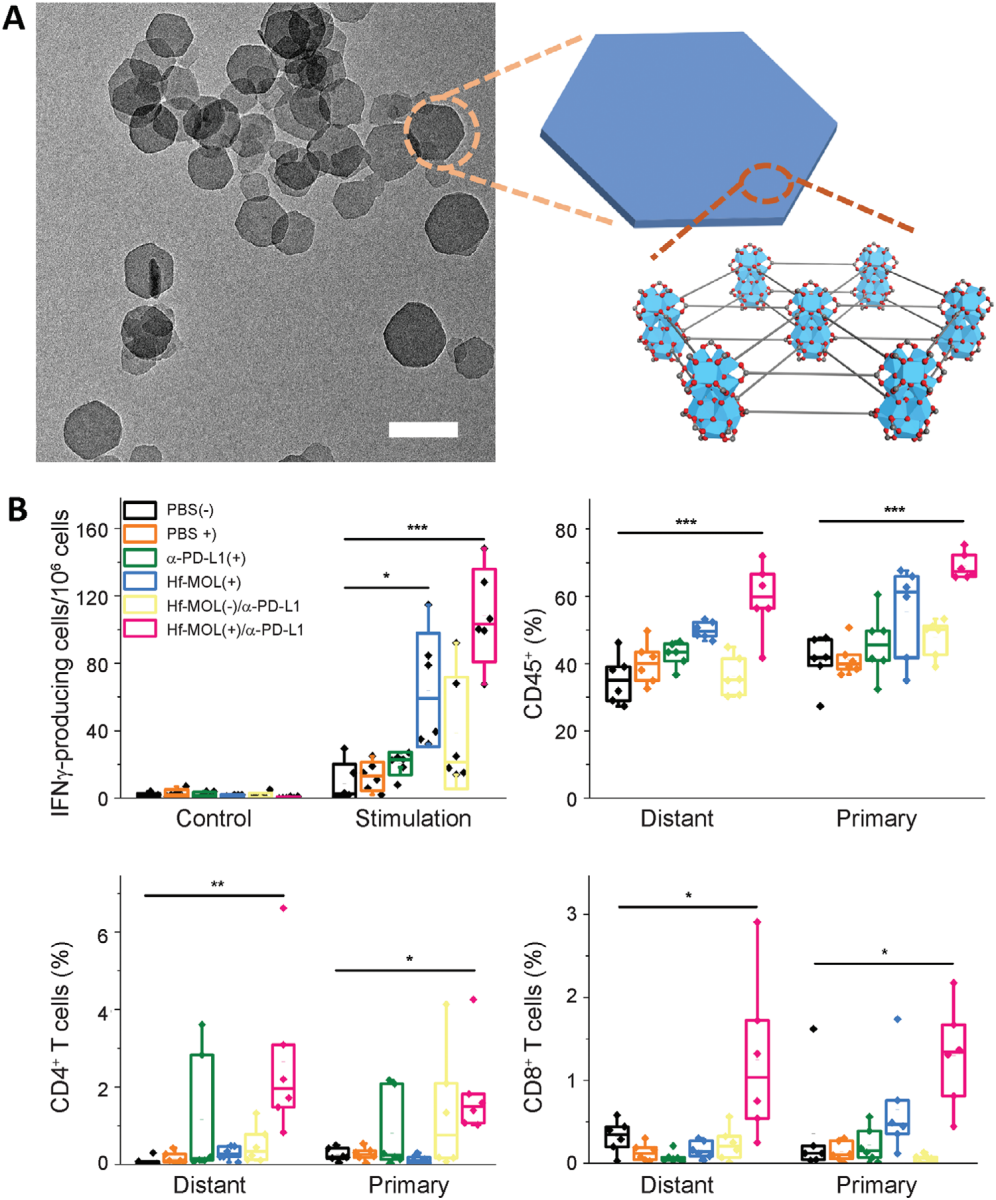
A) TEM image, topological structure, and high‐resolution TEM image of Hf‐MOL. The 2D topology can enable the ready diffusion of ROS into cell organelles. B) Hf‐MOL, when combined with radiation and anti‐PD‐L1 blockades, generated significantly better tumor‐specific T‐cell response than anti‐PD‐L1 alone. Furthermore, it recruited greater number of tumor‐infiltrating B cells and natural killer cells into both primary and distant tumors. Reproduced with permission.^[^ 72 ^]^ Copyright 2019, Elsevier.

The Lin group achieved further improvements to the production of ROS during radiation using nMOF.^[^ 71 ^]^ In their following work, Ni et al. developed an ultrathin nanoscale metal‐organic layer (nMOL) that yields a higher radiosensitization effect than nMOF.^[^ 72 ^]^ Unlike nMOF's 3D crystalline nanoplate morphology, nMOL exhibited 2D, thin, plate‐like morphology, which facilitated the diffusion of generated ^1^O_2_ under radiation (**Figure** **3**A). In combination with anti‐CTLA‐4 antibodies, robust abscopal effects driven by reactivating T cells and inhibiting immunosuppressive myeloid‐derived suppressor cells (MDSCs) were observed in both orthotopic tumors and metastatic lesions (Figure 3B).

In addition to the development of better radioenhancers, the use of multimodal nanoparticles capable of reversing tumor hypoxia and delivering immunostimulatory molecules was also explored. For example, local delivery of radioisotope iodine‐131 was enabled by an in situ‐formed, alginate‐based hydrogel.^[^ 73 ^]^ Catalase and oligodeoxynucleotides containing cytosine‐phosphate‐guanosine sequence (CpG) were incorporated to facilitate the production of ROS under hypoxic tumor microenvironment upon radiation and to elicit a proinflammatory immune response through TLR9 receptor activation. This hydrogel system's improved tumor retention profile achieved an excellent local tumor‐killing effect on a liver rabbit tumor model at low radioactive doses. Furthermore, when combined with anti‐CTLA‐4 antibodies, significant inhibition of tumor metastasis and recurrence was realized. In their following work, the same group provided mechanistic insight into the use of multimodal nanoparticles in improving radiosensitization.^[^ 74 ^]^ Increased effector memory T cells and elevated levels of IL‐12p70, IFN‐γ, and TNF‐α were attributed to a superior antitumor immune response.

Collectively, these preclinical results suggest that nanoparticles are promising platforms for maximizing the immunogenic effect of radiotherapy while limiting the radiation dose. These nanoparticles represent new opportunities in cancer treatment and may foster the development of safer and more effective radioimmunotherapy regimens to treat cancer.

## 5 Ultrasound‐Responsive Nanoparticles to Potentiate Immunotherapy

Despite the great success of phototherapies in preclinical models, translation has often been difficult due to poor tissue penetration, which leads to low therapeutic efficiency. In contrast, the ultrasound‐based therapeutic modality is emerging due to its high tissue‐penetrating capability and controllability.^[^ 75 ^]^ Ultrasound (US) is defined as a type of mechanical sound wave with a periodic vibration at frequencies higher than the human hearing (20 kHz).^[^ 76 ^]^ It can exert various bioeffects, ranging from mechanical liquefaction to vascular damage.^[^ 77 ^]^ We show 3 examples with distinct mechanisms that function in combination with checkpoint blockade: Peritumoral vascular damage elicited by ultrasound and microbubble (USMB) treatment, ROS‐mediated tumor cell killing, and immunogenicity enhancement, and thermal ablation of tumors achieved by high intensity focused ultrasound (HIFU).

To investigate the potential for synergy between a US‐mediated antivascular effect and PD‐1 blockade, Bulner et al. first intraperitoneally administered 200 µg of PD‐1 blockade, applied octafluoropropane‐based microbubbles 30 min later, then followed with 2 min of US at 50 pulses that were 0.1 ms long and spaced 1 ms apart.^[^ 78 ^]^ For the USMB treatment, Bulner et al. reported 88 ± 3.6% blood flow shutdown in tumors, which was much higher than MBs alone. In the CT26 tumor model, USMB + PD‐1 blockade treatment resulted in significantly longer times to reach the survival endpoint, with 1 out of 6 mice exhibiting complete tumor regression. Though the USMB + PD‐1 blockade regimen improved efficacy, the results were far from desirable.

In a rabbit model reported by Wei et al., alterations in blood perfusion, specifically with respect to microvessels, were not observed in the USMB‐treated group.^[^ 79 ^]^ Wei et al. proposed an inverse relationship between tumor permeability and the size of the particulates. USMB could increase the permeability in the whole tumor tissue, especially in the peripheral region, for particulates within the size range of 5–20 nm.

**Figure 4:**
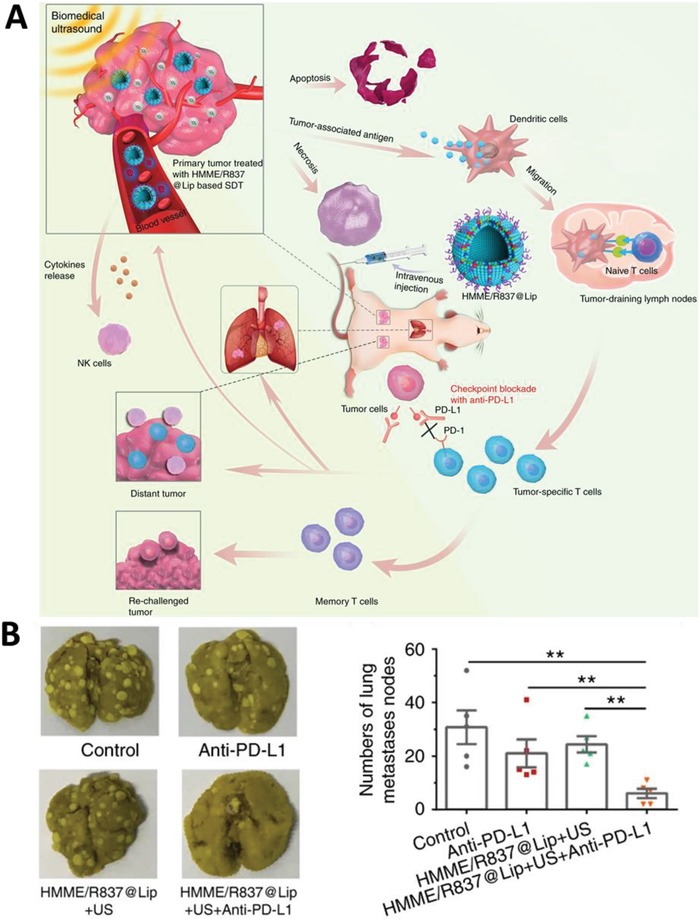
A) Schematic illustration of antitumor immune responses induced by combined SDT with immune‐adjuvant‐contained nanosonosensitisers and checkpoint blockade for effective cancer immunotherapy. B) The combination of HMME/R837@Lip + US and anti‐PD‐L1 blockade resulted in the greatest reduction in the number of lung metastasis nodules. Reproduced with permission.^[^ 12a ^]^ Copyright 2019, Springer Nature.

Yue et al. formulated a liposomal nanoplatform that contained a sonosensitizer, hematoporphyrin monomethyl ether (HMME), and TLR agonist R837 (**Figure** **4**A).^[^ 12a ^]^ An electron spin resonance (ESR) and 1,3‐diphenylisobenzofuran (DPBF) assay were used to qualitatively and quantitatively monitor the US‐mediated ^1^O_2_ production. Upon combining with PD‐L1 blockade, HMME/R837@Lip‐augmented SDT resulted in 95% eradiation of the primary 4T1 tumor as well as 83% eradiation of the distant tumor, which was a significantly better result than anti—PD‐L1 alone. Additionally, no abnormality was observed regarding body weight or serum biochemistry, which supports the biosafety merit of SDT. Furthermore, the combined anti—PD‐L1/SDT regimen achieved a substantial reduction in the 4T1 lung metastases model (Figure 4B). Robust immune activation was attributed to the superior performance of anti‐PD‐L1/SDT, where an increase in CD8^+^ T cells, and NK cells, and a decrease in CD4^+^Foxp3^+^ T_reg_ cells, were observed.

HIFU thermal ablation therapy could achieve synergy with adjuvant and ICB and achieve a profound antitumor effect. Liu's group conducted a study combining HIFU with R837 or a TLR‐4 agonist, monophosphoryl lipid A (MPLA) encapsulated in PLGA nanoparticles, and an anti—CTLA‐4 antibody.^[^ 80 ^]^ The lack of CD4^+^ and CD8^+^ T‐cell infiltration and the presence of abundant CD45^+^CD11b^+^Gr‐1^+^ MDSCs were observed in the distant tumor after treatment of primary tumors by thermal ablation alone. After combining with PLGA‐R837 or PLGA‐MPLA adjuvants, a significant increase in the percentage of matured CD11c^+^CD80^+^CD86^+^ DC was achieved compared to those treated with HIFU alone. The study further incorporated anti‐CTLA‐4 antibody to enhance the effective T‐cell response and inhibit T_reg_ cells. Collectively, HIFU + adjuvant + anti‐CTLA‐4 antibody resulted in the regression of both primary and distant tumors in a CT26 model. A significantly increased CD8^+^/T_reg_ ratio, accompanied by an increase in effector T memory cells, were attributed to the long‐term immune memory against the tumor.

### 5.1 The Clinical Landscape of Ultrasound Therapies with Immunotherapy

These preclinical, ultrasound‐based, combinatorial therapies show great synergy with ICB, yet clinical advancement is still in its infancy. In fact, most of the clinical evaluation of ultrasound concentrates on its utility as a drug delivery enhancer.^[^ 81 ^]^ The lack of clinical advancement of ultrasound‐assisted immunotherapy is primarily due to the lack of fundamental understanding regarding the exact pharmacokinetics/pharmacodynamics mechanisms behind the observed therapeutic efficacy. The difficulty of discriminating the thermal and ROS effects from the mechanical effect poses additional challenges in the clinical advancement of ultrasound, which requires further investigation.

## 6 Magnetic‐Responsive Nanoparticles to Potentiate Immunotherapy

In general, there are two mechanisms behind the use of magnetic‐responsive nanoparticles: magnetic navigation and magnetic hyperthermia (MHT). Magnetic navigation is widely used for tumor‐selective accumulation of superparamagnetic iron oxide (IO) nanoparticles, thereby greatly enhancing intratumoral drug content and reducing systemic side effects.^[^ 82 ^]^ The advancement in surface modification further enabled the incorporation of immunomodulatory modality into IO, thereby complementing immune therapy. MHT therapy is an emerging technology that aims to treat the tumor with heat generated by magnetic nanoparticles under a strong alternating magnetic field (AMF).^[^ 83 ^]^ Due to AMF's deep tissue penetration capability, it may serve as an optimal alternative to PDT. In a clinical setting, effective MHT ablation often requires a highly concentrated local injection of superparamagnetic iron oxide nanoparticles (SPIONs) combined with high‐power AMF. This is due to SPIONs' poor magnetic heating efficiency.^[^ 84 ^]^ Advancement in nanomedicine has enabled a further improvement in MHT; when combined with ICB, a significant efficacy boost was often reported.

Chiang et al. designed fucoidan and aldehyde‐functionalized dextran‐modified IO, IO@FuDex. As previously reported, fucoidan's immunostimulatory functions enhance adaptive immune responses.^[^ 85 ^]^ The presence of aldehyde‐functionalized dextran enabled the immobilization of anti‐PD‐L1 and anti‐CD3/anti‐CD8 antibodies on IO@FuDex via reductive amination. Chiang et al. hypothesized that in situ expansion of tumor‐infiltrating T cells could be achieved with the administration of IO@FuDex^3^, which contains both anti‐PD‐L1 and anti‐CD3/anti‐CD8 antibodies, whereas IO@FuDex^1^ contains anti‐CD3/anti‐CD8 antibodies and IO@FuDex^2^ contains anti‐PD‐L1 antibodies.^[^ 85 ^]^ Magnetic navigation successfully promoted tumor accumulation of IO@FuDex^3^, resulting in over 15% ID g^−1^ at 24 h postinjection, compared to 5% without a magnet or <10% for IO@FuDex with a magnet. The improved tumoral accumulation translated into greater tumor inhibition, which was evidenced by a significant enhancement in survival in both 4T1 and CT26 tumor models. IO@FuDex^3^'s ability to reverse immunosuppressive tumor microenvironment (TME) was noted where CD3^+^CD8^+^and CD3^+^CD4^+^ T‐cell population was significantly enhanced, and T_reg_ was reduced.

To improve SPIONs' poor magnetic heating efficiency, Chao et al. developed pure iron nanoparticles (FeNPs) with high magnetic saturation intensity.^[^ 86 ^]^ After the surface is coated with PEG/dopamine (DA) cografted polymer, FeNPs can be stored as a lyophilized powder for months and used later after being redispersed. The magnetic heating efficiency was evaluated concerning other magnetic nanoparticles, including iron oxide nanoparticles (IONPs), iron oxide nanoclusters (IONCs), and commercially available iron oxide nanoparticles (USPIO‐30). Notably, the temperature of the FeNP‐PEG sample at 2 mg mL^−1^ increased by 45 °C within 5 min—drastically higher than the 0.5, 6.6, and 13.3 °C temperature increases for IONPs, USPIO‐30s, and IONCs, respectively. The demonstrated high magnetic heating efficiency of FeNP‐PEG showed complete ablation of 4T1 tumors after intratumoral administration, even with low heating power (*H*_appl_ × *f*_appl_ = 1.2 × 109 A m^−1^ s^−1^, compared to traditionally used >3 × 109 A m^−1^ s^−1^). Furthermore, with the aid of a neodymium magnet (NE036), tumor‐focused magnetic targeting was achieved upon IV injection, resulting in complete ablation of 4T1 tumors. After combining FeNP‐PEG with R837 and CTLA‐4 checkpoint blockade, the significant abscopal effect was achieved in which both the primary and secondary distant tumors disappeared about one week later in CT26 bilateral models. Furthermore, 80.7% of CD8^+^ T cells polarized into effector T memory cells in the spleen of mice treated with combinatorial therapy—much higher than 16.9% in mice undergoing primary tumor surgery—highlighting the success trigger of systemic immunity. Immune suppression was hampered, which was evidenced by a reduced T_reg_ cell population.

**Figure 5:**
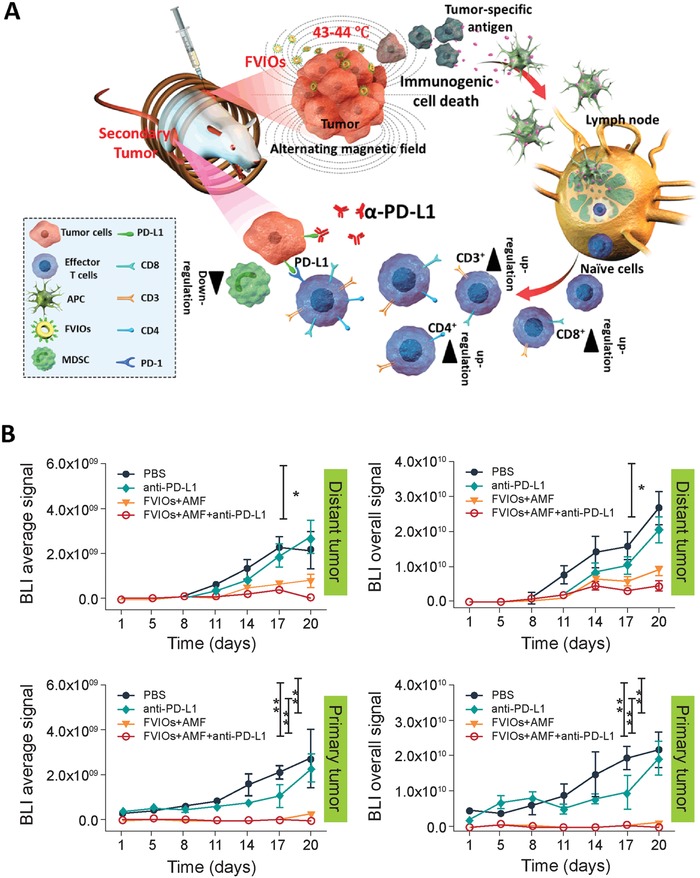
A) The proposed mechanism of FVIO‐mediated mild magnetic hyperthermia plus immune checkpoint therapy activates the antitumor immune response. B) FVIO‐mediated mild magnetic hyperthermia therapy sensitizes tumors to immune checkpoint therapy and results in regression in both primary and distant tumors. Reproduced with permission.^[^ 87 ^]^ Copyright 2019, American Chemical Society.

A similar study was conducted by Liu et al., where PEGylated ferrimagnetic vortex nanoring (FVIO) with efficient heat induction due to a vortex‐to‐onion magnetization reversal process that was used for inducing local hyperthermia under AMF (**Figure** **5**A).^[^ 87 ^]^ Successful MHT‐induced tumor cell death was confirmed in 4T1, where early apoptosis (38.42%) and late apoptosis (48.96%) were reported after AMF exposure for 5 min. Furthermore, a calreticulin (CRT) signal that promotes phagocytic uptake of cancer cells by the immune system was upregulated in the FVIO plus AMF group, suggesting FVIO‐mediated hyperthermia induces tumor immunogenic properties. In combination with anti‐PD‐L1, FVIO‐mediated hyperthermia completely ablated the primary 4T1 tumor with no recurrence up to 32 d after treatment, achieved meaningful abscopal effect, and successfully prevented lung metastasis (Figure 5B).

Magnetic hyperthermia has been incorporated into PDT to potentiate anti‐CTLA‐4 antibody performance. Wang et al. designed Ce6 loaded mesoporous organosilica nanoparticles with Fe_3_O_4_ magnetic heads (M‐MONs@Ce6).^[^ 88 ^]^ To enhance tumor targeting and immune‐evading properties, M‐MONs@Ce6 particles were further cloaked with cancer cell biomimetic vesicles derived from MCF7 breast cancer cells, which resulted in CM@M‐MONs@Ce6. Significant ICD was achieved by CM@M‐MONs@Ce6 with laser and alternating‐current magnetic field evidenced by an increase in CRT‐positive cells and high mobility group box 1 protein (HMGB1) release. An in vivo biodistribution study revealed that the cell membrane camouflaged enhanced tumor accumulation and decreased enrichment in the reticuloendothelial (RES) system. Regression of primary MCF7 tumor and complete eradication of 4T1 lung metastasis were reported when combined with an anti‐CTLA‐4 antibody, providing a promising strategy for multimodal cancer treatment.

### 6.1 The Clinical Landscape of Magnet Therapies with Immunotherapy

Despite great preclinical proof‐of‐concept, we are unaware of any clinical assessment of magnetic drug delivery vehicles combined with ICB.^[^ 89 ^]^ The gap between the lab production of such multimodal nanoparticles and their upscaling synthetic production drastically limits their clinical translation. Furthermore, detailed studies on the degradation and excretion mechanisms are required to introduce such an approach to medical practice. Lastly, in addition to carrier optimization, there is also a need to optimize the design and control of external magnets.

## 7 Major Challenges in Translation and Future Scope from an Industry Perspective

There are several major challenges to overcome when considering how to leverage academic research into novel drug delivery systems for oncology applications toward the integration of such delivery systems into an industrial project portfolio. The general challenges in achieving preclinical to clinical translation have already been discussed in detail and, clearly, a lack of confidence in that translation significantly increases the risk and complexity of delivery technology selection.^[^ 99 ^]^ However, this is a general phenomenon that is common to both academic and industry environments. When specifically considering the translation of preclinical work from academic settings or small startups to a larger industrial setting, some additional challenges need to be considered.

### 7.1 Misalignment of Academic Research and Industrial Interest

Often preclinical work will be conducted using well‐established payloads, particularly chemotherapeutics such as paclitaxel and doxorubicin.^[^ 100 ^]^ While these agents can be successful in generating data that support a method of delivery to the tumor, it is common that the delivery of such a payload is not a high priority for companies that are active in discovering the next generation of oncology therapeutics.^[^ 101 ^]^ This is a complicated issue to resolve because access to more current payloads of interest is not feasible unless more effective academic‐industrial collaborations facilitate the use of novel delivery systems with novel therapeutics. However, the combination of the two clearly increases the risk profile of the research; hence the selection of technologies to evaluate in this manner can be a constraint.

In a similar fashion to the payloads above, there is also often a disconnect between models used in academic research labs and models of high interest within the industry, since human cancer immune biology is not always well reflected in the models that are typically used in academic research labs.^[^ 102 ^]^ This is partly driven by the fact that the choice of model and payload are often considered together. Even when the models used are aligned, there is often a set of nuances in the application of that model that can make results interpretation more challenging. These considerations include tumor placement, tumor size upon initiation of treatment, and dosing volumes and frequency. Differences here can again lead to difficulties in making firm conclusions about data presented that would warrant the commitment to investigate a new technology internally.

Besides, it can often be challenging to build sufficient controls within a single study to provide a clear and unambiguous benefit from the delivery technology under investigation.^[^ 103 ^]^ Often a lack of resources leads to incomplete study design, making it difficult to quantify potential improvements in efficacy. Prof. Warren Chan highlights an excellent example of the difficulties associated with the interpretation of literature toward the identification of a delivery technology worthy of investment.^[^ 7a ^]^

As noted in prior sections, there are many challenges and risks associated with the exploration and potential adoption of novel delivery technology. For these reasons, the introduction of such technologies is often focused on therapeutics that have previously been validated in a clinical setting. In these cases, the challenge then becomes demonstrating the benefits of the new delivery system versus the original and presumably more straightforward delivery approach. In areas such as the delivery of chemotherapeutics using targeted nanoparticles, the heterogeneity of disease in larger patient populations is often cited as a difficult challenge to overcome.^[^ 104 ^]^

However, it is likely that in an industry setting, companies that do not have significant capabilities toward molecular design are more likely to generate the most interest and possess the required level of risk tolerance toward drug delivery. In an effort to maintain lower chemistry, manufacturing and controls (CMC) risk posture, companies that have invested significant amounts in their molecular design capabilities—either in medicinal chemistry or protein sciences groups—will often favor a strategy where attempts will be made to solve challenges via molecular design. Regardless, such environments do create opportunities where delivery technology and molecular design can be used in harmony with a “design for delivery” strategy in which chemical matter and a particular delivery technology have parallel optimization.^[^ 105 ^]^

A lot of the challenges listed above are difficult to resolve when academic research and industry adoption of research remain 2 separate endeavors. The most effective solution is to enhance strategic collaborations between research groups and industry by structuring them in a way that allows for many of the gaps in terms of tool compounds, in vivo study design, etc., to be better aligned toward the interest and current portfolios of industry. This can allow for the generation of much more meaningful, quantifiable results that can act as a catalyst for the prioritization of novel delivery technologies for clinical validation.

### 7.2 Considerations for Chemistry, Manufacturing, and Controls

Numerous nanoconstructs have demonstrated utility in preclinical models for external‐stimuli‐enhanced ICB, as summarized in the above sections. They can be classified into delivery platforms such as polymeric micelles, hydrogels, liposomes, dendrimers, and inorganic nanoparticles. Of these platforms, the most observable success is for liposome‐based nanomedicines, which are evident from the preclinical/clinical efforts as well as approved nanomedicine in general.^[^ 106 ^]^ For the translation of these constructs to a clinical or commercial product, consideration for large scale productions related to reproducibility, infrastructure, technology, and cost needs to be taken into account. With the approved liposomal formulations, there are several publications as well as industrial‐scale production facilities with wide‐ranging expertise available within their manufacturing network.^[^ 107 ^]^ However, some of these complex nanoconstructs, with co‐delivery or self‐assembly of external stimuli agent and ICB, are currently limited to bench scale. Also, analytical tools to evaluate the impact of physical characteristics of nanoconstructs on the in vivo performance, such as particle size, size distribution, surface charge density, etc., need to be in place. The required analytical tools vary based on the construct and add to the complexity of commercialization. The regulatory guidelines are also evolving, which present unanticipated challenges in taking these constructs to approval. There is a need for separately defined regulations; the current guidelines are not appropriate for assessing the quality, safety, and efficacy of these nanoconstructs for clinical use.^[^ 108 ^]^

While it is often appropriate for researchers to be unconstrained in their design by potential issues in the CMC space, the lack of a line of sight or a perceived high CMC complexity can also be a barrier to more widespread adoption or exploration of a novel delivery system.^[^ 109 ^]^ This is especially true when considering factors in the previous sections, that make it difficult to understand or quantify the potential benefits fully. Platforms requiring complex and multistep synthesis can be very challenging to manufacture on a large scale.^[^ 110 ^]^ Each step creates challenges in understanding the control of impurity removal, raw material quality, and the impact on the final critical quality attributes of the nanoconstruct drug product. The impact of the various parameters—including size, morphology, porosity, and storage stability of the final product—are important considerations, along with the impact on the efficacy of external stimuli responsiveness. Variations in batch‐to‐batch quality can potentially lead to changes in physicochemical properties, pharmacokinetic parameters, and pharmacodynamics interactions. Since these nanoparticles can aggregate in the bloodstream, they need to be both stable upon storage and clinical administration.^[^ 111 ^]^ Platforms like inorganic nanoparticles have increased research interest, but the evidence regarding toxicity is conflicting; toxicity has shown to be highly dependent on size and morphology.^[^ 112 ^]^ Additionally, the route of administration (e.g., intratumoral vs intravenous) may vary significantly between in vivo mice models and human models, which may necessitate optimizing the characteristics of the constructs to meet the target product profile.^[^ 113 ^]^

While designing these constructs, it is crucial to critically evaluate the value of each added layer of complexity from the perspective of stability, efficacy, and safety with the final route of administration in mind. As a delivery system becomes more complex in its design, so do the various factors related to the control that will become increasingly important when developing and scaling up a robust process. A lack of precedent will create increased scrutiny on the process control strategy.^[^ 114 ^]^ Novel excipients will require substantial attempts to eliminate or minimize safety risks. These elements all contribute to an overall higher risk profile for development that would need to be layered on top of the already significant risks associated with drug discovery and development.

### 7.3 Adoption and Translation to Marketed Therapies

Oncology is a very active therapeutic area with more than 23 indications now approved, and more than 60 new active substances (many for multiple tumors) approved over the last 5 years.^[^ 115 ^]^

While new active substances are being developed and registered, the overall response and survival of cancer patients remain far from ideal. Pairing immunotherapies with chemotherapy, targeted therapy, and radiation therapy to drive improved outcomes continues to be a pursuit.^[^ 116 ^]^ Moreover, new modalities such as oncolytic viruses, cancer vaccines, cell therapies, new immunomodulators, and bispecific antibodies to further improve target engagement, tumor cell kill, immune upregulation, and tumor infiltration, and subsequent responses are being evaluated in clinical trials.^[^ 117 ^]^ As these approaches materialize, there remains an opportunity for alternative methods to improve the therapeutic index at the recommended doses of established standards of care.

One of the approaches to improve the therapeutic index has been tumor‐targeting to reduce systemic exposure and achieve localized concentrations in the tumor. Various tumor‐targeted therapies have been evaluated over the last several decades. These targeted approaches include passive, active, and external‐stimuli‐responsive approaches. While passive targeting (e.g., particle‐based approaches leveraging EPR in tumors) has been an active stream resulting in multiple drug products in the marketplace (Doxil, AmBisome, Abraxane), it has also lacked precision and patient‐to‐patient consistency in delivery. Active targeting, on the other hand, has the potential to direct the active agent into the tumor tissue by leveraging tumor‐specific receptors/antigens. However, such differential expression and uniqueness of the target are incredibly challenging. These challenges have driven evaluation of physically assisted (external stimuli) approaches that have demonstrated the complementarity of existing standards of care as exemplified in sections above. While the therapeutic effectiveness of known anti‐cancer drugs could be enhanced using external stimuli (light, thermal, magnetic, ultrasound, etc.), there exist translational challenges from preclinical to clinical proof‐of‐concept and then to an unequivocal demonstration of superiority to the relevant comparator.

A case in point is the recent failure of Halozyme's lead program—a phase 3 study in pancreatic cancer—where PEGPH20 (a PEGylated version of Halozyme's hyaluronidase enzyme) was tested with Celgene's Abraxane and chemo drug gemcitabine. The PEGPH20 enzyme was aimed at improving the drug penetration in solid tumors, such as pancreatic tumors after intravenous administration, thus resulting in improved treatment outcomes. The enzyme combination with chemotherapy did not extend patients' lives compared to Abraxane and gemcitabine alone.^[^ 118 ^]^ While these external‐stimuli‐based interventions have shown promise preclinically and in small clinical trials, their transition into marketed interventions/therapies remains challenging because of inherent variability in the patient population and an evolving pharmaco‐ and biotherapeutic landscape, which continues to raise the bar and create an upward shift in endpoints demonstrating noninferiority. Establishing these external stimuli approaches in tumor types (e.g., glioblastomas, pancreatic) and in patients who do not respond well to available therapy could be a way of balancing the risk/benefit and enabling clinical advancement. Generation of systematic delivery systems and data sets across in vitro, preclinical in vivo, and clinical studies will be required to reduce the number of variables under evaluation at any given time.

## Conflict of Interest

The authors declare no conflict of interest.
